# The Efficacy and Safety of Fostamatinib in Elderly Patients with Immune Thrombocytopenia: A Single-Center, Real-World Case Series

**DOI:** 10.1155/2022/8119270

**Published:** 2022-11-03

**Authors:** Jessica Liu, Cyrus C. Hsia

**Affiliations:** Department of Medicine, Division of Hematology, London Health Sciences Centre, London, ON, UK

## Abstract

Fostamatinib is a small molecule spleen tyrosine kinase (Syk) inhibitor that was approved for the treatment of adult patients with immune thrombocytopenia (ITP) in second-line therapy. Syk inhibition prevents cytoskeletal rearrangements during phagocytosis, allowing platelet survival in ITP. However, fostamatinib treatment in elderly patients with ITP has not been well established. We performed a retrospective review of all elderly patients (age greater than or equal to 65 years) who had started on fostamatinib for the treatment of ITP at a single tertiary care centre to evaluate its efficacy and safety. Seven patients, median age 80 years (range 78–94), four women and three men, all of Caucasian background, with various comorbidities, started fostamatinib 100 mg orally twice daily as second or subsequent line therapy. Patients had a diagnosis of ITP for a median of 6 years (range approximately 6 months–30 years), had six comorbidities (range 2–14), and experienced 2 unique prior lines of ITP therapy (range 1 to 6). Over 1290 days of fostamatinib exposure, two patients required dose escalation to 150 mg orally twice daily, while five patients remained on the initial starting dose of 100 mg twice daily. The median platelet count at the time of initiating fostamatinib was 25 × 10^9^/L (range less than 10–193). The median time to response (defined as any first platelet count greater than or equal to 30 × 10^9^/L) was 19 days (range 0–181 days), with two patients responding rapidly (5 days and 19 days). Two patients required dose escalation and rescue therapy, and these same two patients discontinued fostamatinib after 175 days and 216 days of treatment. Treatment was tolerated in all patients with no thromboembolic events observed. One death was noted and unrelated to treatment. Overall, fostamatinib was effective and safe for the majority of these very elderly patients with ITP.

## 1. Introduction

Immune thrombocytopenia (ITP) is an autoimmune disorder characterized by decreased platelet counts and variable bleeding manifestations. It is mediated by autoantibodies binding to platelets, resulting in phagocytosis and platelet destruction by splenic macrophages and impairment of megakaryocyte function, resulting in decreased platelet production [1]. First-line therapy for ITP consists of corticosteroids with or without intravenous immunoglobulin (IVIG). However, many patients require additional treatment due to nonresponse, relapse, or intolerance. There is currently no consensus on second-line therapies for ITP, which include medical therapies such as rituximab, thrombopoietin receptor agonists (TPO-RAs), and immunosuppressants, and surgical management with splenectomy [2]. Due to limited evidence from randomized control trials, these second-line therapies are typically selected based on patient and provider preference, with current guidelines recommending shared decision-making [3].

Fostamatinib is a small molecule spleen tyrosine kinase (Syk) inhibitor that was approved for the treatment of ITP by the US FDA in 2018. The Syk pathway is activated upon the binding of the Fc region of autoantibodies to Fc-gamma receptors on macrophages, leading to cytoskeletal rearrangements during phagocytosis [4]. Syk has also been demonstrated to play a role in platelet activation in thrombosis through glycoprotein VI (GPVI) and C-type lectin-like receptor-2 (CLEC-2) signalling; it has therefore been suggested that fostamatinib may reduce the risk of thromboembolic events without impacting hemostasis [5].

The efficacy of fostamatinib in adults with chronic ITP has been established in phase 3 clinical trials [6], and further studies have established fostamatinib as an effective second-line treatment [7]. Common adverse effects of this medication include diarrhea, hypertension, nausea, and transaminitis [6]. The risk of thrombosis has been shown to be low, including in patients with additional thrombotic risk factors [5].

However, studies investigating fostamatinib as a treatment for ITP in patients over the age of 65 years are limited. Furthermore, elderly patients are typically underrepresented in clinical trials, with phase 3 clinical trials of fostamatinib having been conducted in a cohort with a median age of 54 years [6]. Currently, dose adjustments of fostamatinib are not recommended for elderly patients, as the pharmacokinetics of recommended fostamatinib dosages are not altered to a clinically relevant degree by age [8]. Despite this, elderly patients present unique challenges in the management of ITP, including multiple comorbidities, decreased functional status, and increased risk of toxicities, which can complicate treatment selection and response.

## 2. Methods

We performed a retrospective review of all elderly patients who had started on fostamatinib for the treatment of ITP at a single tertiary care centre, the London Health Sciences Centre (LHSC). Eligible patients were 65 years or older, had ITP as defined by the American Society of Hematology criteria [3], and received fostamatinib as part of routine care. Patients who received fostamatinib as part of a clinical trial were excluded. Data included patient demographics, timing, and dosing of treatment with fostamatinib, a list of other treatments, platelet counts, and reported adverse events and outcomes of patients.

### 2.1. Statistical Analysis

Descriptive statistics were used to characterize the population. Continuous variables were summarized using means and standard deviations (SD) or medians with ranges as appropriate. All analyses were conducted using Microsoft Excel.

## 3. Results

Seven patients, median age of 80 years (range 78–94), four women and three men, all of Caucasian background, with various comorbidities, started fostamatinib as second or subsequent line therapy (Table 1). These individuals had a median of six comorbidities (range 2–14), with a median of two cardiovascular risk factors (range 1–5), a mean weight of 69.4 kg (±7.4), a mean height of 167 cm (±11.3), and a mean body mass index of 25 kg/m2 (±3.2). The median age of patients at the time of diagnosis of ITP was 73 years (range 63–94) and patients had a diagnosis of ITP for a median of 6 years (range approximately 6 months–30 years). They had a median of 2 unique prior lines of ITP therapy (range 1 to 6), including corticosteroids (prednisone and/or dexamethasone), intravenous immunoglobulin, splenectomy, rituximab, azathioprine, and thrombopoietin receptor agonists. All patients had exposure to either prednisone or dexamethasone or both; four patients received intravenous immunoglobulins; one patient had a splenectomy; three had azathioprine; one had rituximab; and two had a thrombopoietin receptor agonist (Table 1). Two patients developed intolerance to a previous treatment due to adverse effects of the medication (patient 1 to corticosteroids and patient 4 to azathioprine).

**Figure 1:**
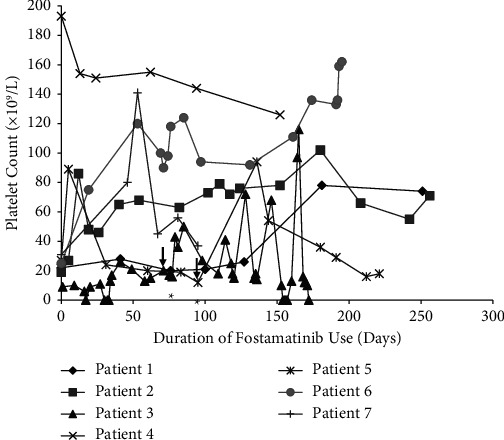
Platelet response to fostamatinib use over time. All patients were started on a dose of 100 mg orally twice daily. A dose increase to 150 mg twice daily was required in patient 3 and patient 5, denoted by the vertical arrow (↓). The administration of rescue therapy with either dexamethasone only (patient 3) or dexamethasone and IVIG (patient 5) is denoted by the asterisk (*∗*).

Fostamatinib 100 mg orally twice daily was started between June 2021 and January 2022 and increased at the treating physician's discretion based on response up to 150 mg orally twice daily. Two patients required the dose escalation while the remaining five patients remained on the original 100 mg twice-daily dosing since the last assessment. The median platelet count at the time of initiating fostamatinib was 25 × 10^9^/L (range less than 10–193) (Table 2). Three patients had persistent ITP and four had chronic ITP at treatment initiation. The median time to response (defined as any first platelet count greater than or equal to 30 × 10^9^/L) was 19 days (range 0–181 days), with two patients responding rapidly (patient 5 in 5 days and patient 6 in 19 days). Median peak platelet counts during the observation period were 129 × 10^9^/L (range 94–193), and median platelet counts at the latest investigation were 74 × 10^9^/L (range 0–162). Two patients required dose escalation and rescue therapy, and these same two patients discontinued fostamatinib after 175 days and 216 days of treatment. One patient, study patient 3, died from numerous complications from infections and worsening congestive heart failure that were unrelated to this treatment. One patient, study patient 5, stopped fostamatinib due to relapsed disease after 216 days. The remaining five patients continue on fostamatinib 100 mg orally twice daily with no dose adjustments required, with four individuals maintaining a partial response (platelets greater than or equal to 30 × 10^9^/L) and one individual maintaining a complete response (platelets greater than or equal to 100 × 10^9^/L) at the time of last assessment. Study patient 4 demonstrates the challenges with regional access to ITP therapies as she lost funding to her TPO-RA and was transitioned to fostamatinib with a platelet count of 193 × 10^9^/L and was able to maintain a complete response (platelets > 100 × 10^9^/L). Another patient, study patient 7, started with a platelet count of >30 × 10^9^/L as he had a number of relapses with platelets <20–30 prior to the start of fostamatinib, requiring courses of dexamethasone with transient partial responses. Fostamatinib was obtained and started soon after one of these courses of dexamethasone 40 mg daily × 4 days. Treatment was tolerated in all patients with no thromboembolic events observed. Transaminitis (patient 2) and petechial rash (patients 3 and 5) were the only adverse effects observed (Table 2). Platelet response to fostamatinib use over time is shown in Figure 1.

## 4. Discussion

To date, this is the largest case study investigating the successes and challenges of fostamatinib use in this elderly ITP population. ITP is a heterogeneous disease that is further complicated in elderly patients by medical comorbidities and the risk of medication toxicity. Currently, corticosteroids are the recommended first-line therapy for ITP, including for elderly patients, although in this population, special attention must be paid to adverse effects including steroid-induced diabetes, muscle weakness, and osteoporosis. The adverse effects of established second-line therapies are varied. Rituximab has an associated risk of infections secondary to B-cell depletion; one study found this risk to be acceptable, with the 3 episodes of fatal infection occurring in older adults, two of whom had severe comorbidities [9]. TPO-RAs such as eltrombopag are associated with an increased risk of thromboembolism (GlaxonSmithKline, 2015), on top of the increased risk of thromboembolic events seen in elderly patients with ITP [10]. Real-world TPO-RAs (eltrombopag and romiplostim) use in older patients was effective but associated with a 15.6% recurrent thrombosis risk [11]. Oral TPO-RA, avatrombopag, was recommended for an elderly ITP patient in the review by [12]. In the elderly population, splenectomy is associated with an increased risk of relapse and an increased risk of perioperative complications, including major bleeding, and it is often contraindicated due to the comorbidities seen in this population [13, 14]. Furthermore, accessibility to various therapies such as TPO-RA may be limited geographically, including our own local practice. The elderly patients in our region are greatly disadvantaged due to access to these ITP treatments.

Fostamatinib represents a novel treatment option that has been shown to produce durable responses in those who have failed the aforementioned first- and second-line therapies for ITP [6]. In this case series, the cumulative patient-years of ITP was approximately 77 years, and all seven patients we observed had thrombotic risk factors and were started on fostamatinib after failing at least one prior line of ITP treatment. Fostamatinib was started as a second-line therapy in 2 patients (6 and 7) and as a third-line or subsequent therapy in 5 patients. There was a total of 1290 days of fostamatinib exposure in these seven patients combined.

The safety profile of fostamatinib seen here is consistent with that demonstrated in phase 3 clinical trials, in which both transaminitis and rash were noted as adverse effects of the medication [6]. Interestingly, the two most common adverse effects in these clinical trials, namely diarrhea and hypertension, were not seen among the 7 cases presented here. The absence of thromboembolic events in these cases is also consistent with previous literature, where it has been suggested that the Syk-inhibitory mechanism of fostamatinib may interfere with platelet activation in thrombosis via GPVI and CLEC-2 [5]. In keeping with the current recommendations [3], dose reduction was not required in any of the patients despite their advanced age.

## 5. Conclusion

Treatment selection for elderly patients with ITP poses unique challenges, and there is a relative paucity of literature addressing this population. As demonstrated in the cases presented here, fostamatinib may represent a safe and effective therapy in ITP for elderly patients who have previously failed at least one prior line of treatment. One limitation of this case series is the relatively short duration of time on fostamatinib. Furthermore, given its recent approval and introduction as a treatment option for ITP, further studies examining real-world cases of fostamatinib use in elderly patients would be helpful in clarifying the safety profile of fostamatinib in this population.

## Figures and Tables

**Table 1:** Baseline characteristics.

Patient	Weight (kg), height (cm), BMI (kg/m2)	Number of comorbidities, number of cardiovascular and thrombotic risk factors	Age at ITP diagnosis (years)	Type of ITP at start of fostamatinib	Steroid exposure, IVIG, splenectomy, other previous ITP treatments	Prior unique treatments	Duration of ITP diagnosis prior to fostamatinib (years)
1 94F Caucasian	64, 167, 22.9	6 1	72	Chronic	Dex and pred, no, no, azathioprine	2	22.6
2 94M Caucasian	63.4, 158, 25.4	11 3	94	Persistent	Dex, yes, no, none	2	0.2
3 80F Caucasian	64.6, 166, 23.4	14 5	80	Persistent	Dex and pred, yes, no, none	2	0.5
4 78F Caucasian	77, 155, 32.0	3 3	73	Chronic	Pred, yes, no, azathioprine, TPO-RA	4	5.4
5 78M Caucasian	70, 169, 24.5	4 2	63	Chronic	Dex and pred, yes, yes, azathioprine, rituximab, TPO-RA	6	14.7
6 94F Caucasian	65, 165, 23.9	6 1	65	Chronic	Pred, no, no, none	1	29.8
7 80M Caucasian	82, 190, 22.7	2 1	80	Persistent	Dex and pred, no, no, none	1	0.4

Dex, dexamethasone; pred, prednisone; TPO-RA, thrombopoietin receptor agonist; cardiovascular and thrombotic risk factors including known thrombosis, coronary artery disease, strokes, or transient ischaemic attacks, hypertension, diabetes, dyslipidemia, and smoking.

**Table 2:** Characteristics on fostamatinib.

Patient	Platelets at fostamatinib start (×10^9^/L)	Platelet response (platelets ≥ 30 × 10^9^/L)	Time to platelet response (days)	Peak platelet count (×10^9^/L)	Most recent platelet count (×10^9^/L)	Rescue therapy on fostamatinib	Adverse events	Remains on fostamatinib at most recent assessment	Died	Fostamatinib exposure (days)
1	20	Yes	181	140	74	No	No	Yes	No	252
2	19	Yes	0	102	75	No	Transaminitis	Yes	No	214
3	0	No	81	116	0	Dex	Petechial rash, mild transaminitis	No	Yes	175
4	193	Yes	0	193	126	No	No	Yes	No	152
5	27	Yes	5	94	18	Dex and IVIG	Petechial rash	No	No	216
6	25	Yes	19	162	162	No	No	Yes	No	188
7	31	Yes	46	141	37	No	No	Yes	No	93

Dex, dexamethasone; IVIG, intravenous immunoglobulin.

## Data Availability

Data are available upon request to investigators.
